# Single nucleotide polymorphisms and the risk of developing a second primary cancer among head and neck cancer patients: a systematic literature review and meta-analysis

**DOI:** 10.1186/s12885-021-08335-0

**Published:** 2021-06-02

**Authors:** Ilda Hoxhaj, Vladimir Vukovic, Stefania Boccia, Roberta Pastorino

**Affiliations:** 1 Section of Hygiene, University Department of Life Sciences and Public Health, Università Cattolica del Sacro Cuore, Rome, Italy; 2 Department of Woman and Child Health and Public Health - Public Health Area, Fondazione Policlinico Universitario A. Gemelli IRCCS, Rome, Italy; 3 Center for Disease Control and Prevention, Institute of Public Health of Vojvodina, Novi Sad, Serbia

**Keywords:** Head and neck cancer, Second primary cancer, Single nucleotide polymorphism, Personalized medicine, Biomarker

## Abstract

**Background:**

Head and Neck Cancer (HNC) survivors are at increased risk of developing a second primary cancer (SPC). Along with the environmental risk factors, genetic factors have been associated with a potential increased susceptibility to SPC development. We aim to identify the Single Nucleotide Polymorphisms (SNPs) that contribute to SPC development among HNC survivors through a systematic review and meta-analysis.

**Methods:**

We searched PubMed, Scopus and ISI Web of Science for eligible studies published in English until January 31st, 2020. We included studies reporting primary data that evaluated the association between SNPs and SPC risk in HNC patients. Data were pooled in a random-effect meta-analyses, when at least two studies on the same SNP evaluated the same genotype model. Heterogeneity was assessed using the χ2-based Q-statistics and the I^2^ statistics. Quality of the included studies was assessed using the Q-Genie tool.

**Results:**

Twenty-one studies, of moderate to good quality, were included in the systematic review. Fifty-one genes were reported across the included studies to have significant associations with an increased SPC risk. Overall, 81 out of 122 investigated SNPs were significantly associated with the SPC risk. Seven studies were included in the meta-analysis, which showed five SNPs associated with an increased risk of SPC: p21C70T, CT + TT (HR = 1.76; 95% CI: 1.28–2.43); FASLG -844C > T, CT + TT (HR = 1.82; 95% CI: 1.35–2.46), P21 C98A, CA + AA (HR = 1.75; 95% CI: 1.28–2.38); FAS -670A > G (HR = 1.84; 95% CI: 1.28–2.66) and GST-M1, Null genotype (HR = 1.54; 95% CI: 1.13–2.10).

**Conclusions:**

The identified SNPs in our systematic review and meta-analysis might serve as potential markers for identification of patients at high risk of developing SPC after primary HNC.

**PROSPERO Registration Number:**

CRD42019135612.

**Supplementary Information:**

The online version contains supplementary material available at 10.1186/s12885-021-08335-0.

## Background

Head and neck cancer (HNC) is a group of cancers that occur in oral cavity, pharynx and larynx, it is the sixth most common cancer, with approximately 650.000 new cases and 300.000 deaths annually [1]. Although the overall survival-rate after the primary HNC have increased [2] due to an improved diagnosis and therapeutic approaches, the incidence of second primary cancer (SPC) remains one of the main long-term causes of mortality [3, 4]. It is estimated that approximately 15–25% of HNC patients develop SPCs within five years of initial diagnosis [5, 6]. The main risk factors associated with the development of SPC in HNC survivors include environmental factors such as tobacco use, alcohol consumption, and human papillomavirus infection, as well as genetic factors [1]. With advancements in genetics and genomics, especially with DNA sequencing, genetic factors have become increasingly studied for their potential role in the development of HNC [7]. The affected genes are responsible for normal cell growth, DNA-repair, cell-cycle control, programmed cell death (apoptosis), cell differentiation and oxidative stress [8]. Genome wide association studies (GWAS) have already demonstrated important associations between several genetic abnormalities and HNC carcinogenesis [9, 10]. Although single nucleotide polymorphisms (SNPs) have been widely studied for a potential increased susceptibility to HNC development [11, 12], there is still no consistent evidence about the effect of SNPs among HNC patients into developing SPCs. Identification of genes and genetic markers associated with poor survival after HNC may distinguish patients with increased SPC risk, facilitating surveillance and enabling targeted interventions while reducing mortality [13]. Therefore, it is important to identify pathways of carcinogenesis that might serve as potential markers for identification of patients at high risk of developing SPC after primary HNC. To address this issue, we aimed to review and analyze the available literature and identify the SNPs that contribute to SPC development among HNC patients and to provide quantitative assessment of the associations between SNPs and SPC risk.

## Methods

This systematic review was conducted and reported based on the Preferred Reporting Items for Systematic Reviews and Meta-analysis Protocol (PRISMA-P) checklist [14] (Additional file 1). The protocol of this research was registered on International prospective register of systematic reviews database (PROSPERO) with the registration number CRD42019135612.

### Eligibility criteria

Studies were considered eligible for inclusion if they evaluated the association between SNPs and SPC risk in HNC patients, and if they provided the effect measures with the corresponding 95% confidence interval (CI). Studies reporting primary data were included. There were no restrictions on primary HNC stage or treatment status. According to Warren and Gates criteria, a SPC is defined as a second cancer that developed after a primary cancer, that is of non-squamous cell origin, or which has developed in a different location other than the primary cancer. If the second cancer is of squamous cell origin and has developed in the same region as the primary cancer, it is only coded as an SPC if more than 60 months had passed since the primary cancer diagnosis [15]. Therefore, outcomes such as recurrences, metastases or multiple primary cancers were excluded from this review. Studies were also excluded if they reported genetic alterations such as microsatellite instability or genetic variants over-expression. Editorials, comments, conference papers, narrative reviews, case reports, case series and descriptive cross-sectional studies were also excluded.

### Search strategy

Two researchers [IH; VV] systematically searched PubMed, Scopus and ISI Web of Science online databases for eligible studies published in English from inception until January 31st, 2020. The following search query was used in PubMed: (((Head and Neck) OR pharynx OR pharyngeal OR oropharynx OR oropharyngeal OR hypopharynx OR hypopharyngeal OR nasopharynx OR nasopharyngeal OR larynx OR laryngeal OR (oral cavity) OR (upper aerodigestive tract) OR UADT) AND (tumor OR neoplasm* OR cancer OR malignanc* OR carcinoma)) AND ((second primary) OR SPC) AND ((genetic AND (characterization OR alterations OR variant OR polymorphism)) OR gene OR microRNA OR SNP OR (single nucleotide polymorphisms) OR polymorphism* OR biomarker*).

Other two databases were searched using the appropriately modified PubMed search query (details are available upon request).

Subsequently, the reference lists of the included studies were manually searched for additional relevant publications. In the second step, aiming to understand whether GWASs identified any genetic loci associated with the risk of SPC in HNC patients, we also explored the following GWAS databases: GWAS Central National Human Genome Research Institute (NHGRI GWAS Catalog) [16], The database of Genotypes and Phenotypes (dbGaP) [17]; The GRASP: Genome-Wide Repository of Associations Between SNPs and Phenotypes, and The genome wide association database (GWAS DB) [18].

### Study selection

Identified studies from all databases were uploaded to Mendeley Reference Manager and duplicate articles were removed. Two independent researchers [IH; VV] performed the first screening based on titles and abstracts. In the second stage of screening, studies with full texts available were carefully reviewed. Studies that met the eligibility criteria were selected for inclusion. The PRISMA flow chart was created, reporting all the steps of search strategy and study selection: total number of studies retrieved, number of excluded studies during title/abstract screening, and number of studies excluded during full-text assessments, along with reasons of exclusion. Disagreements were resolved through discussion with the third researcher [RP] until the consensus was reached.

### Data extraction

From each of the included studies, two researchers [IH; VV] independently extracted the following data: first author, year of publication, study design and setting, study size, patients' ethnicity, primary HNC site, follow-up period, number of patients with SPC, SPC site(s), genes, chromosomes, SNPs, measure of association and corresponding genetic model. Researchers double-checked the extracted data and few subsequent discrepancies were resolved through discussion and in consultation with another researcher [RP].

### Quality assessment

Two researchers [IH; VV] independently assessed quality of the included studies using the Q-Genie tool, which was specifically designed for the evaluation of genetic association studies [19]. This tool contains 11 items, assessing: rationale for conducting the study, selection and definition of outcome of interest, selection and comparability of comparison groups, technical and non-technical classification of the exposure, other source of bias, appropriateness of sample size and power, description of the analyses and statistical methods used, testing of assumptions and appropriateness of inferences drawn from results. Each item is rated on a 7-point scale: “1 point – poor”; “2 and 3 points - good”; “4, 5 and 6 points - very good” and “7 - excellent”. For studies with control group, the overall quality of studies is categorized as the following: “poor quality” if score ≤ 35; “moderate quality” if score > 35 and ≤ 45; and a “good quality” if score > 45. For studies without control groups, values for each of the categories listed are ≤ 32; > 32 and ≤ 40; and > 40, respectively. Any disagreement was solved through discussion with the third researcher [RP].

### Data synthesis and analysis

The main findings were reported in a tabular synthesis, separately for each SNP, and the qualitative synthesis reported possible associations of each SNP with the SPC risk. Meta-analysis was performed considering different study designs, and the SNPs studied reported in each study. When at least two studies on the same SNP were available and evaluated the same genotype model, the data were pooled in a random-effect meta-analyses [20]. Effect size were expressed as hazard ratios (HR) or odds ratios (OR) with the corresponding 95% confidence intervals (CI), as appropriate. We stratified the analyses according to the site of SPC. The heterogeneity between studies was assessed using the χ2-based Q-statistics and the *I*^2^ statistics [21]. The heterogeneity was considered low if the *I*^2^ value was < 25%. *P*-values of less than 0.05 were considered statistically significant. To assess the presence of publication bias (where appropriate), we conducted Egger’s asymmetry test (level of significance *p* < 0.05) for the SNPs with at least three pooled studies [22]. Statistical analyses were performed using the Stata software package version 13 (StataCorp. College Station. Texas).

## Results

### Search results

**Fig. 1:**
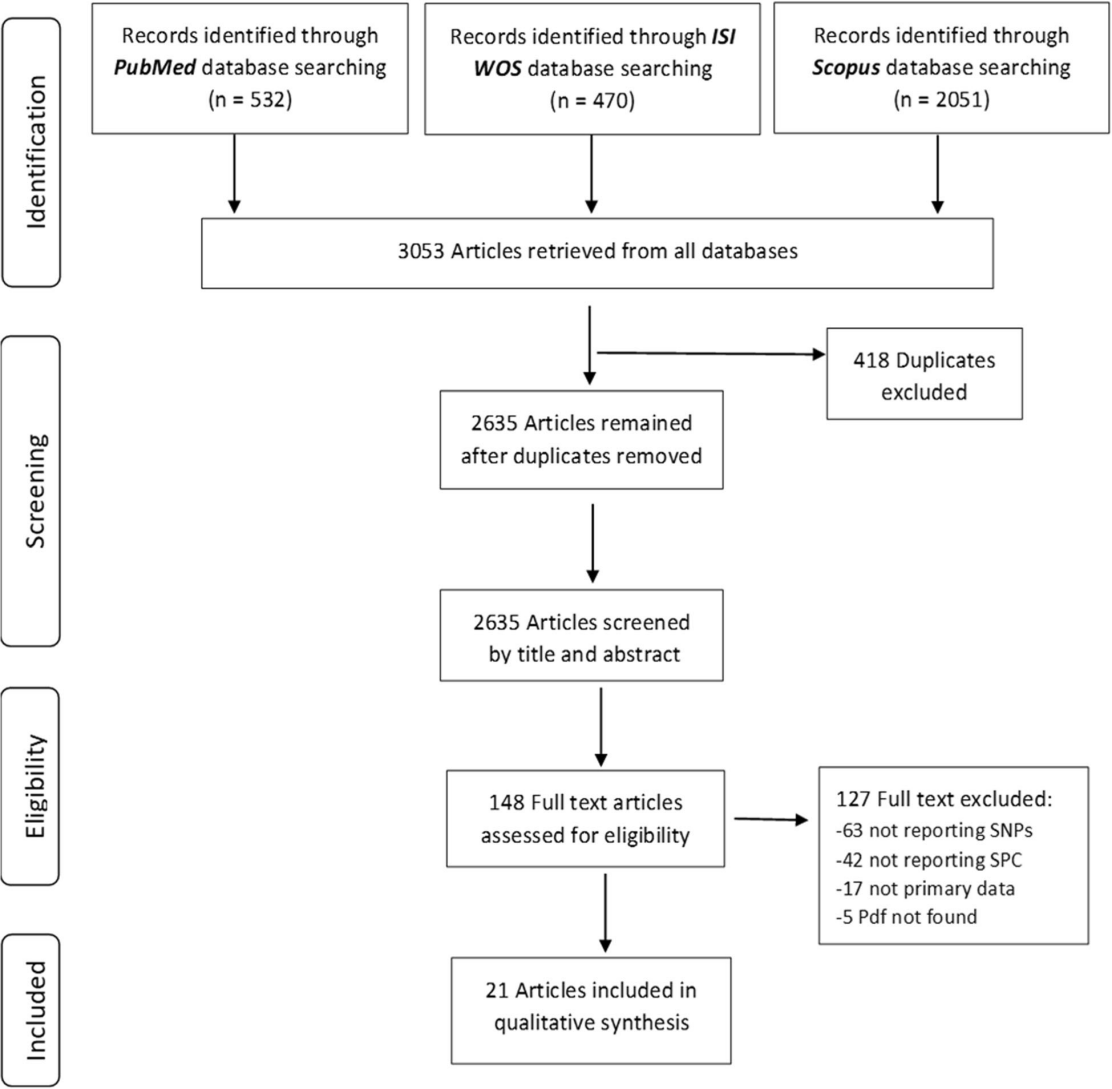
PRISMA Flow chart of screening and selection process of studies included in systematic review

The initial search of PubMed, ISI Web of Science and Scopus databases identified a total number of 3053 articles. After removing the duplicates, 2635 articles were screened by title and abstract. One hundred forty-seven full-text articles were evaluated, of which twenty-one articles met the inclusion criteria. No additional studies were included after checking the reference lists of the included articles. The entire process of the literature search and study selection is reported in details in the PRISMA Flowchart in Fig. 1. From the search of GWAS databases, we did not find any GWAS on genetic loci associated with a risk of SPC in HNC patients.

### Characteristics of the studies

Characteristics of the twenty-one included studies [23–43] are reported in Table 1. Sixteen studies were of cohort study design and five were case-control studies, published from 2005 to 2019. Eighteen studies were conducted in the USA, seventeen of which retrieved patients from the same randomized placebo-controlled trial [44], investigating different genes and SNPs. The remaining three studies were performed in Canada, United Kingdom and Italy. The number of HNC patients included in the cohort studies varied from 215 to 1529. The majority of patients were male. Median follow-up time varied from 2.1 to 5.21 years. Studies conducted in the USA reported data on the ethnicity where most patients were of non-Hispanic white ethnicity. Regarding primary HNC site, twenty studies evaluated all HNC sites (oral cavity, larynx, pharynx), whereas the study by Gal et al. [28] investigated only oral cavity site. As for the SPC site, twelve studies reported data on tobacco-related SPC (esophagus, lung or bladder) and on non-tobacco-related SPC (prostate, thyroid or colon); while nine remaining studies did not report site-specific data.

**Table 1:** Characteristics of the twenty-one studies included in systematic review

First author, Year [Ref]	Country	Study design; Study period	Number of HNC patients	Follow-up, years ^a) mean (SD), b) median [IQR]^	Patients' age, years ^a) mean (SD); b) median [IQR] ^	Gender, Male (%)	Ethnicity (%)	Primary HNC site (%)	Primary HNC stage (%)	Number of patients with SPC (%)	Site of SPC	Chromosome	Genes investigated	SNP
Li F, 2010 [23]	USA, Texas	Cohort;1995–2007	1.271 HNC	2.2^b)^ [0–11.8]	at HNC 57.2 ^a)^; at SPC 60.9 ^a)^	75.6	Non-Hispanic white (85.4)	oropharyx (45.2); non-oropharynx (54.8)	I or II (25.9); III e IV (74.1)	109 (8.6)	42 HNC; 38 other tobacco related (head & neck, esophagus, lung, bladder); 29 other non-tobacco related	17	p53	rs1042522
Wang J, 2010 [24]	USA, Texas	Case-control; 1991–1999	147 HNC SPC cases; 293 controls	cases 2.3^b^; controls 5.0^b^	61.15 ^a)^ (10.25)	79.55	Caucasian (96.1)	larynx (47.6); oralcavity (31.29); pharynx (21.09)	I (59.2); II (40.8)	147 (100)	NR	1	RGS2; GS13; RGS8; GS11; RGS5; RGS3; RGS7	rs2179653; rs3795617; rs6670735; rs739999; rs11586945; rs3747813; rs6689169
Lee JJ, 2011 [35]	USA, Texas	Cohort;1991–1999	215 HNC	NR	60.9 ^a)^ (10.57)	76.28	Caucasian (5.8)	larynx (56.7); oral (31.6); pharynx (11.6)	I (66.1); II (33.9)	76 (35.3)	NR	9; 11	RXRA; JAK2; TNKS1BP1; MMP3; RAD54L; BCCIP; SLC31A1; GSTM5; TSC1; JAK2; CDK8; TSC1; FLT3; CA9; TSC1; MP21	rs3118570; rs1887427; rs3025090; rs12137934; rs6596428; rs11244664; rs2233913; rs11101992; rs7040593; rs2274471; rs1410280; rs3827665; rs9551427; rs1243872; rs11602501; rs739442; rs10901431
Li F, 2009 [37]	USA, Texas	Cohort; 1995–2007	1.384 HNC	2.2^b)^ [0–11.8]	at HNC 57.3 ^a)^; at SPC 60.8 ^a)^	76.1	Non-Hispanic white (83.8)	oral cavity (32.7); oropharynx (45.1); hypopharynx/larynx (22.2)	I or II (25.4); III or IV (74.6)	110 (8.0)	77 tobacco related, 29 non-tobacco related, 4 both tobacco- and non-tobacco related	1	P73	G4C14-to-A4T14 (rs2273953 and rs1801173)
Zafereo, 2009 [39]	USA, Texas	Cohort; 1995–2006	1.376 HNC	2.2^b)^	at HNC 57.3 ^a)^; at SPC 60.8 ^a)^	76.0	Non-Hispanic white (84.0)	oral cavity (32.9); oropharynx (44.9); larynx/ hypopharynx (22.2)	I or II (25.5); III or IV (74.5)	110 (8.0)	43 HNC; 38 tobacco-related; 29 non-tobacco related	9	ERCC1; XPA; XPC; XPD; XPG	ERCC1 rs3212986 (C8092A); XPA rs1800975 (G23A); XPC Ala499Val (rs1799793); XPC Lys939Gln; XPD Asp312Asn (rs1799793); XPD Lys751Gln; XPG His1104Asp (rs17655)
Zafereo, 2009 [40]	USA, Texas	Cohort; 1995–2006	1.376 HNC	2.2^b)^	at HNC 57.3^a)^; at SPC 60.8^a)^	76.0	Non-Hispanic white (84.0)	oral cavity (32.9); oropharynx (44.9); larynx/hypopharynx (22.2)	I or II (25.5); III or IV (74.5)	110 (8.0)	43 HNC SPC; 38 tobacco-related; 29 non-tobacco related	1; 22	GST	GSTM1; GSTT1; GSTM1 + GSTT1; GSTP1_105; GSTP1_114
Leoncini E, 2015 [41]	Italy	Cohort; 2001–2012	801 HNC	59 ^b)^ months [1.6–76]	at HNC 62 ^a)^	79.5	NR	oral cavity (23.8); oropharynx (17.4); hypopharynx (5.4); larynx (49.7)	I (25.6); II (22.1); III (18.8); IV (33.5)	117 (14.6)	head and neck, lung	4	ADH1B; ADH7	ADH1B rs1229984; ADH7 rs1573496
Jefferies S, 2005 [42]	England	Case control; NR	61 HNC SPC cases; 259 controls	NR	at HNC 58.9 ^a)^; at SPC 64.4 ^a)^	37Male SPC	NR	oral cavity (46); oropharynx (13); hypopharynx (8); larynx (33)	NR	61 (100)	HNC; prostate; ovary, breast, lung, colon; stomach; bladder; blood, melanoma	3	GPX1	GPX1 Polymorphisms: GPX1 ALA5; GPX1 ALA6; GPX1 ALA7
Lei D, 2010 [43]	USA, Texas	Cohort; 1995–2007	1.282 HNC	2.84^b)^ [0–11.8]	at HNC 57.4^a)^; at SPC 60.8 ^a)^	at HNC 76.1; at SPC: 23.9	Non-Hispanic white (84.5)	oral cavity (32.4); oropharynx (44.7); hypopharynx/larynx (22.9)	I or II (25.2); III or IV (74.8)	120 (9.4)	any SPC	6	p21	p21 C98A (rs1801270); p21 C70T (rs1059234)
Lei D, 2010 [25]	USA, Texas	Cohort; 1995–2007	1.286 HNC	2.47^b)^ [0–11.8]	at HNC 57.5^a)^; at SPC 60.8 ^a)^	at HNC 76.0; at SPC 24.0	Non-Hispanic white (84.5)	oral cavity (32.4); oropharynx (44.6); hypopharynx/larynx (23.0)	I or II (25.2); III or IV (74.8)	120 (9.3)	any SPC	10; 1	FAS; FASL	FAS − 1377 (G > A) (rs2234767); FAS -670A > G (rs1800682); FASLG -844C > T (rs763110); FASLG -124A > G (rs5030772)
Guan X, 2013 [26]	USA, Texas	Case control; 1999–2007	1.066 HNC SPC cases; 1.074 controls	NR	Cases 57.1^a)^ (11.2); Controls 56.7 ^a)^ (11.0)	Cases: 75.3 Controls: 76.3	Non-Hispanic White (100)	oropharynx (50.7); non-oropharynx (49.3)	NR	49 (5.8)	NR	4	CASP3	rs1049253 T > C
Zhang Y, 2012 [27]	USA, Texas	Cohort; 1995–2007	1.269 HNC	2.17^b)^ [0–11.8]	57 ^b)^	75.6	Non-Hispanic white (85.4)	oral cavity (31.9); oropharynx (45.2); hypopharynx/larynx (22.9)	I or II (25.9); III or IV (74.1)	109 (8.6)	NR	4	p53; p73	p53 codon 72; p73 G4C14-to-A4T14
Gal TJ, 2005 [28]	USA,Washington	Cohort; 1988–1995	279 HNC	NR	at HNC 54^a)^ (8.6)	71.3	Caucasian (92.8)	oralcavity (100)	NR	85 (30.5)	lung and trachea, larynx, oral cavity, oropharynx, esophagus	19; 14; 10	XRCC1; XRCC3; XPD; MGMT	XRCC1 Arg 399 Gln; XRCC3 Thr 241 Met; XPD Lys 751 Gln; MGMT Leu84Phe; MGMT Val143Ile
Sun Y, 2016 [29]	USA, Texas	Cohort; 1995–2010	1.529 HNC	4.27^b)^	at OPC 54 ^b)^ [28–84]; at non-OPC: 59 ^b)^ [18–94]	77.5	Non- Hispanic white (87.2)	oropharynx (49.2); non-oropharynx (50.8)	I or II (25.2); III or IV (74.8)	42 OPC (5.6); 82 non-OPC (10.9)	NR	10; 1	FAS; FASL	rs1800682; rs2234767; rs5030772; rs763110
Wang Z, 2012 [30]	USA, Texas	Cohort; 1995–2007	1.292 HNC	2.8^b)^ [0–11.8]	at HNC 57.4 ^a)^	76	Non-Hispanic white (84.6)	oral cavity (32.5); oropharynx (44.5); larynx/hypopharynx (23.0)	I or II (25.3); III or IV (74.7)	120 (9.3)	81 tobacco related; 35 non-tobacco related; 4 both tobacco and non-tobacco related	12; 6	CDKN1B; P21	rs2066827; rs1801270; rs1059234
Zhang Y, 2011 [31]	USA, Texas	Cohort; 1995–2007	1.287 HNC	2.47^b)^ [0–11.8]	at HNC 57^b)^ [18–94]	75.9	Non-Hispanic white (84.6)	oral cavity (32.3); oropharynx (44.6); larynx/hypopharynx (23.1)	I or II (25.2); III or IV (74.8)	120 (9.3)	HNC, lung, esophagus, bladder, blood, prostate, colon, thyroid, breast, kidney, liver, endometrium, maxillary sinus	9	p14ARF	rs3731217; rs3088440
Jin L, 2013 [32]	USA, Texas	Cohort; 1995–2007	1.283 HNC	2.83^b)^ [0.2–11.8]	at HNC 57 ^b)^	76.0	Non-Hispanic white (84.6)	oral cavity (32.4); oropharynx (44.6); larynx/hypopharynx (23.0)	I or II: (25.2); III or IV: (74.8)	120 (9.3)	tobacco related: esophagus, lung, bladder; non- tobacco related: prostate, thyroid, colon	17; 1; 9; 12;	p53; p73; p14ARF; MDM2; MDM4	p53 codon 72 (rs1042522); rs2273953; rs3731217; rs3088440; rs2279744; rs937283; rs11801299; rs1380576; rs10900598
Azad AK, 2012 [33]	Canada	Cohort;1994–2000	531 HNC	5.12^b)^	63 ^b)^ [33–86]	79	NR	oralcavity (12); pharynx (5); larynx (83)	Stage I (62.0); Stage II (38.0)	111 (21)	lung, head and neck, prostate, skin, colorectum kidney, breast, esophagus, bladder, blood, hypothalamus.	1; 2; 5; 9; 11; 13; 14; 16; 17; 19; 22;	CCND1; TP53; DNMT3B; ERCC1; ERCC2; ERCC4; ERCC5; MSH2; XPA; XRCC1; XRCC3; FGFR4; CTLA4; MMP3; CYP2D6	rs603965; rs678653; rs1042522; rs2424913; rs3212986; rs1799793; rs13181; rs1799801; rs1047768; rs17655; rs2303426; rs1800975; rs25487; rs861539; rs351855; rs231775; rs35068180; rs35742686; rs3892097
Wu X, 2009 [36]	USA, Texas	Nestle Case-control; 1991–1999	150 HNC SPC cases; 300 controls	NR	Cases 61.40 ^a)^ (10.2); Controls 61.05 ^a)^ (10.18)	80	Caucasian (99.7)	larynx (47); oral (32); pharynx (21)	I (59.0); II (41.0)	150 (100)	NR	10; 2; 7	MKI67; NHEJ1; CDK6;TNFRSF10B; MNAT1; GSTM4; GLI2; RNF2; CAT; PROM1; IGF1R; CFTR; AXIN1; CHFR; GSTM4; KRAS; MRC2; BRCA2; PDGFB	rs12359892; rs359974; rs7781436; rs876435; rs12888332; rs506008; rs7561607; rs6684195; rs17387169; rs7168671; rs7591; rs2306536; rs7118388; rs9622978; rs2237724; rs604337; rs3826537; rs9562605; rs2300181; rs11047917
Minard CG, 2006 [34]	USA, Texas	Cohort;1991–2006	303 HNC	4^a)^	at HNC 62.5 ^a)^ (10.6)	83.8	Caucasian (100)	larynx (65.7); oral (23.8); pharynx (10.6)	I (67.0); II (33.0)	50 (16.5)	head and neck pharynx, larynx, lung, esophagus, bladder, kidney, pancreas, prostate	1; 22	GST-M1; GST-T1	GST-M1 null genotype; GST-T1 null genotype
Zhang X, 2010 [38]	USA, Texas	nested case–control; 1991–1999	150 HNC SPC cases; 300 controls	NR	Cases 61.4 ^a)^ (10.21); Controls 61.05 ^a)^ (10.18)	80	Caucasian (99.7)	larynx (58.4); oral (28.8); pharynx (12.7)	I (63.5); II (36.5)	150 (100)	NR	22	SMC1B; BCL2L2; GSTM3; IL1R1; NR1I2; SSTR2; SUFU; RAN; XPO5; RNASEN	rs3747238; rs1884056; rs15864; rs3917328; rs3732360; rs7210080; rs11594179; rs11061209; rs2227301; rs699937; rs7735863; rs6884823; rs3792830; rs669702; rs639174; rs3805500; rs7719666; rs17410035

*Abbreviations: NR* not reported, *HNC* head and neck cancer, *SPC* Second primary cancer; *SD* standard deviation; *IQR* interquartile range; *OPC* oropharyngeal cancer; *SNP* Single nucleotide polymorphisms

Fifty-one genes (*p53, GST, p73, p21, ERCC5, MDM4, MMP21, GSTM5, BCCIP, TNKS1BP1, RAD54L, CAD9, CDK8, FAS, JAK2, P27, SLC31A1, DNMT3B, MDM2, P14ARF, MMP3, RXRA, TSC1, CDC25C, FASL, FLT3, CASP3, KRAS, RGS5, BCL2L2, GPX1, MKI67, MNAT1, RNASEN, IGF1R, PDGFB, TNFRSF10B, CDK6, AXIN, XPO5, CAT, GLI2, CFTR, RGS11, GSTM4, IL1R1, NR1I2, NHEJ1, SMC1B, SSTR2, RNF2*) were reported to have significant associations with the SPC risk. Overall, the most investigated genes were *GST gene family* (five studies), *p53* (four studies), *p73* (three studies), and by 2 studies each of the following genes *p21, FAS* and *FASL,* and *XPD (ERCC2).* A total of 122 different SNPs were explored, of which 81 SNPs were significantly associated with the SPC risk, in particular with an increased risk. Data for the investigated SNPs are reported separately, according to the study design. The SNPs investigated in cohort studies are reported in Table 2, whereas the SNPs evaluated in case-control in case-control studies are reported in Table 3. The *GST gene family* polymorphisms explored were GST-M1, GST-T1 and GST-P1. GST- M1 null genotype was associated with an increased risk for any SPC (HR = 1.99; 95% CI: 1.11–3.56) and for tobacco-related SPCs (HR = 2.16; 95% CI: 1.01–4.62), whereas the GST-T1 null genotype demonstrated statistically non-significant protective effects for the SPC development (HR = 0.59; 95% CI: 0.25–1.41). Patients with both *GST* non null genotypes were 0.52 times less likely (95% CI: 0.28–0.96) to develop a SPC compared to participants who had the GST-M1 null and GST-T1 non null genotypes. Patients with the *GSTP1* gene Ile105Val polymorphism had a 1.7-fold elevated risk for developing SPC compared to patients with the wild-type genotype (HR = 1.7; 95% CI: 1.1–2.5). The combined risk of *GSTP1* gene 105 and *GSTP1* 114 polymorphisms, increased SPC risk, suggesting that two polymorphisms may have a joint effect on the risk of SPC development [40].

**Table 2:** Associations of SNPs with second primary cancer risk in sixteen cohort studies included in systematic review

SNP	Gene	Chromosome	First author, year [ref]	Genotype	Number of patients (%)	HR	LCI	UCI
G4C14-to-A4T14 (rs2273953 and rs1801173)	p73	1	Li F, 2009 [37]	GC/GC	741 (57.7)	1 (ref.)		
				GC/AT	484 (37.7)	0.61	0.40	0.93
				AT/AT	59 (4.6)	0.44	0.14	1.41
				GC/AT + AT/AT	543 (42.3)	0.59	0.39	0.89
			Zhang Y, 2012 [27]	GA + AA	530 (41.8)	1 (ref.)		
				GG	739 (58.2)	**1.68**	**1.12**	**2.52**
GST-M1	GST	1	Zafereo, 2009 [40]	Wild-type	601 (49.6)	1		
				Null	610 (50.4)	1.4	0.9	2.0
			Azad AK, 2012^a^ [33]	N/A N/A	Absent 274 (52.0); Present 255 (48.0)	1.13	0.78	1.66
			Minard CG, 2007 [34]	Nonnull	154 (50.8)	1		
				Null	149 (49.2)	**1.99**	**1.11**	**3.56**
GSTM1 + GSTT1	GST	1	Zafereo, 2009 [40]	M1 Wild and T1 Wild	470 (38.8)	1		
				Either M1 Null or T1 Null	621 (51.3)	1.3	0.8	1.9
				M1 Null and T1 Null	120 (9.9)	1.6	0.9	3.1
GSTP1_114	GST	1	Zafereo, 2009 [40]	Ala/Ala	1021 (84.4)	1		
				Ala/Val + Val/Val	189 (15.6)	0.8	0.5	1.5
GST-T1	GST	1	Minard CG, 2006 [34]	Nonnull	249 (82.2)	1		
				Null	54 (17.8)	0.59	0.25	1.41
			Zafereo, 2009 [40]	Wild-type	960 (79.3)	1		
				Null	251 (20.7)	1.1	0.7	1.8
			Azad AK, 2012^a^ [33]	N/A N/A	Absent 103 (19.0); Present 427 (81.0)	0.95	0.6	1.51
Leu84Phe	MGMT	10	Gal TJ, 2005 [28]	84 AnyPhe	55 (20.1)	0.53	0.28	1.02
rs1042522	P53	17	Azad AK, 2012^a^ [33]	Arg72Pro C > G	CC 302 (57.0); CG 196 (37.0); GG 32 (6.0)	0.78	0.56	1.09
			Jin L, 2013 [32]	WW	661 (51.5)	1		
				WP/PP	622 (48.5)	**1.6**	**1.1**	**2.4**
			Li F, 2010 [23]	Arg/Arg (ref)	657 (51.7)	1.00		
				Arg/Pro	490 (38.5)	**1.75**	**1.17**	**2.62**
				Pro/Pro	124 (9.8)	0.93	0.44	1.95
				Arg/Pro+Pro/Pro	614 (48.3)	**1.58**	**1.07**	**2.34**
			Zhang Y, 2012 [27]	WW	655 (51.6)	1		
				WP + PP	614 (48.4)	**1.58**	**1.07**	**2.34**
rs1047768	ERCC5	13	Azad AK, 2012^a^ [33]	C581T T > C	CC 200 (38.0); CT 256 (48.0); TT 72 (14.0)	1.08	0.82	1.41
p21 C70T (rs1059234)	p21	6	Lei D, 2010 [43]	CC	1102 (86.0)	1		
				CT	164 (12.8)	**1.92**	**1.21**	**3.05**
				TT	16 (1.2)	1.06	0.26	4.42
				CT + TT	180 (14.0)	**1.82**	**1.16**	**2.85**
			Wang Z, 2012 [30]	CC (Ref.c)	706 (54.6)	1		
				CT + TT	586 (45.4)	**1.7**	**1.1**	**2.7**
rs10900598	MDM4	1	Jin L, 2013 [32]	GT/TT	883 (68.8)	1		
				GG	400 (31.2)	**1.4**	**1.0**	**2.1**
rs10901431	MMP21	10	Lee JJ, 2011 [35]	dominant (common genotype model)	NR	**1.96**	**1.19**	**3.33**
rs11101992	GSTM5	1	Lee JJ, 2011 [35]	dominant (common genotype model)	NR	**2.00**	**1.23**	**3.33**
rs11244664	BCCIP	10	Lee JJ, 2011 [35]	additive (common genotype model)	NR	**1.67**	**1.16**	**2.38**
rs11602501	TNKS1BP1	11	Lee JJ, 2011 [35]	dominant (common genotype model)	NR	**1.96**	**1.19**	**3.23**
rs11801299	MDM4	1	Jin L, 2013 [32]	GG	835 (65.1)	1		
				AG/AA	448 (34.9)	1.1	0.7	1.5
rs12137934	RAD54L	1	Lee JJ, 2011 [35]	additive	NR	**1.85**	**1.23**	**2.78**
rs1229984	ADH1B	4	Leoncini E, 2015 [41]	GG	NR	1		
				GG/GT	NR	0.58	0.28	1.20
rs1243872	CA9	9	Lee JJ, 2011 [35]	dominant (common genotype model)	NR	**1.92**	**1.19**	**3.13**
Lys 751 Gln (rs13181)	XPD (ERCC2)	19	Gal TJ, 2005 [28]	Gln/Gln	37 (13.4)	0.74	0.32	1.72
				Lys/Gln	127 (46.0)	1.19	0.73	1.94
				AnyGln	164 (59.4)	1.09	0.68	1.73
			Azad AK, 2012^a^ [33]	Lys751Gln A > C	TT 241 (45); TG 232 (44); GG 57 (11)	0.91	0.69	1.20
			Zafereo, 2009 [39]	Lys/Lys and Lys/Gln	1126 (88.0)	1		
				Gln/Gln	154 (12.0)	1.1	0.6	1.9
rs1380576	MDM4	1	Jin L, 2013 [32]	CC	547 (42.6)	1		
				CG/GG	736 (57.4)	1.1	0.8	1.6
rs1410280	CDK8	13	Lee JJ, 2011 [35]	dominant (common genotype model)	NR	**4.35**	**1.49**	**12.50**
rs1573496	ADH7	4	Leoncini E, 2015 [41]	CC	NR	1		
				CG/GG	NR	1.25	0.75	2.08
GSTP1_105 (rs1695)	GST	1	Zafereo, 2009 [40]	Ile/Ile	484 (40.1)	1		
				Ile/Val + Val/Val	723 (59.9)	**1.7**	**1.1**	**2.5**
His1104Asp (rs17655)	ERCC5 (XPG)	13	Azad AK, 2012^a^ [33]	His1104Asp G > C	CC 200 (38); CT 256 (48); TT 72 (14)	0.94	0.69	1.28
			Zafereo, 2009 [39]	His/His and His/Asp	819 (95.6)	1		
				Asp/Asp	38 (4.4)	1.1	0.4	2.6
Asp312Asn (rs1799793)	XPD (ERCC2)	19	Azad AK, 2012^a^ [33]	Asp312Asn G > A	GG 243 (46); GA 231 (44); AA 54 (10)	1.05	0.80	1.40
			Zafereo, 2009 [39]	Asp/Asp and Asp/Asn	1086 (87.2)	1		
				Asn/Asn	160 (12.8)	0.7	0.4	1.4
Ala499Val (rs2228000)	XPC	3	Zafereo. 2009 [39]	Ala/Ala and Ala/Val	757 (90.2)	1		
				Val/Val	82 (9.8)	0.7	0.4	1.4
rs1799801	ERCC4	16	Azad AK, 2012^a^ [33]	T2505C T > C	TT 255 (48); TC 226 (43); CC 47 (9)	1.05	0.79	1.39
FAS -670A > G (rs1800682)	FAS	10	Lei D, 2010 [25]	AA	333 (25.9)	1		
				AG + GG	953 (74.1)	**1.57**	**1.00**	**2.54**
				AA ref. (OPC)	7	1.0		
				AG + GG (OPC)	35	2.2	0.9	5.3
			Sun Y, 2016 [29]	AA ref. (non_OPC)	13	1.0		
				AG + GG (non_OPC)	69	**2.4**	**1.1**	**5.1**
rs1800975 (G23A)	XPA	9	Azad AK, 2012^a^ [33]	50UTR A > G	GG 209 (40); GA 262 (50); AA 58 (10)	1.08	0.81	1.44
			Zafereo, 2009 [39]	GG + GA	767 (86.3)	1		
				AA	122 (13.7)	0.7	0.4	1.3
P21 C98A (rs1801270)	P21	6	Wang Z, 2012 [30]	CC (Ref.c)	1.105 (85.5)	1		
				CA + AA	187 (14.5)	**1.7**	**1.1**	**2.6**
			Lei D, 2010 [43]	CC	1095 (85.4)	1		
				CA	168 (13.1)	**1.81**	**1.14**	**2.87**
				CA + AA	19 (1.5)	**1.8**	**1.14**	**2.82**
				AA	187 (14.6)	1.71	0.41	7.03
rs1887427	JAK2	9	Lee JJ, 2011 [35]	dominant (common genotype model)	NR	**2.33**	**1.41**	**3.85**
P27 T109G (rs2066827)	P27	12	Wang Z, 2012 [30]	TT (Ref.c)	706 (54.6)	1		
				TG + GG	586 (45.4)	**2.0**	**1.5**	**3.1**
Lys939Gln (rs2228000)	XPC	3	Zafereo, 2009 [39]	Lys/Lys and Lys/Gln	821 (85.6)	1		
				Gln/Gln	138 (14.4)	0.9	0.5	1.5
rs2233913	SLC31A1	9	Lee JJ 2011 [35]	dominant (common genotype model)	NR	**3.03**	**1.39**	**6.67**
FAS −1377 (G > A) (rs2234767)	FAS	10	Lei D, 2010 [25]	GG	1.023 (79.6)	1		
				GA + AA	263 (20.4)	0.87	0.56	1.36
				GG ref. (OPC-HNC)	34	1.0		
				AG + AA (OPC-HNC)	8	0.8	0.4	1.9
			Sun Y, 2016 [29]	GG ref. (non-OPC-HNC)	64	1.0		
				AG + AA (non-OPC-HNC)	18	1.0	0.5	1.8
rs2273953	p73	1	Jin L, 2013 [32]	GA/AA	746 (58.1)	1		
				GG	537 (41.9)	**1.5**	**1.0**	**2.2**
rs2274471	JAK2	9	Lee JJ, 2011 [35]	additive	NR	**1.79**	**1.18**	**2.70**
rs2279744	MDM2	12	Jin L, 2013 [32]	TT	741 (57.7)	1		
				GT/GG	542 (42.3)	**1.9**	**1.2**	**2.7**
rs2303426	MSH2	2	Azad AK, 2012^a^ [33]	C211þ9G G > C	CC 214 (41); GC 251 (48); GG 56 (11)	0.89	0.67	1.19
Val143Ile	MGMT	10	Gal TJ, 2005 [28]	143 Any Ile	66 (24.2)	1.10	0.66	1.85
rs231775	CTLA4	2	Azad AK, 2012^a^ [33]	A49G A > G	AA 247 (47); AG 221 (42); GG 62 (11)	1.03	0.78	1.37
rs2424913	DNMT3B	20	Azad AK, 2012^a^ [33]	C149T C > T	CC 183 (35); CT 247 (47); TT 96 (18)	**1.49**	**1.15**	**1.95**
Arg 399 Gln (rs25487)	XRCC1	19	Azad AK, 2012^a^ [33]	Arg399Gln A > G	CC 222 (42); CT 235 (44); TT 73 (14)	1.17	0.89	1.54
			Gal TJ, 2005 [28]	Gln/Gln	25 (9.4)	1.56	0.73	3.45
				Any Gln	142 (53.2)	0.87	0.55	1.39
				Arg/Gln	117 (43.8)	0.79	0.48	1.29
rs3025090	MMP3	11	Lee JJ, 2011 [35]	dominant (common genotype model)	NR	**3.57**	**1.64**	**7.69**
rs3088440	p14ARF	9	Jin L. 2013 [32]	GG	1034 (80.6)	1		
				GA/AA	249 (19.4)	**1.6**	**1.1**	**2.4**
			Zhang Y, 2011 [31]	GG (Ref.c)	1038 (80.7)	1.0	1	
				GA	219 (17.0)	**1.69**	**1.11**	**2.56**
				AA	30 (2.3)	1.05	0.33	3.37
				GA + AA	249 (19.3)	**1.61**	**1.07**	**2.43**
rs3118570	RXRA	9	Lee JJ, 2011 [35]	dominant (common genotype model)	NR	**3.33**	**1.67**	**6.67**
rs3212986 (C8092A)	ERCC1	19	Zafereo, 2009 [39]	CC + CA	1000 (93.7)	1		
				AA	67 (6.3)	0.9	0.4	1.9
			Azad AK, 2012^a^ [33]	C8092A C > A	CC 320 (60); AC 179 (34); AA 31 (6)	1.06	0.77	1.46
rs35068180	MMP3	11	Azad AK, 2012^a^ [33]	1612insA −/A	5A/5A 128 (24); 5A/6A 275 (53); 6A/6A 122 (23)	1.01	0.76	1.34
rs351855	FGFR4	5	Azad AK, 2012^a^ [33]	Gly388Arg G > A	CC 281 (53); CT 210 (40); TT 37 (7)	0.98	0.73	1.32
rs35742686	CYP2D6	22	Azad AK, 2012^a^ [33]	DelA	AA 441 (87); GA 63 (12); GG 1 (1)	0.83	0.48	1.43
rs3731217	p14ARF	9	Jin L, 2013 [32]	TT	963 (75.1)	1		
				TG/GG	320 (24.9)	**1.5**	**1.0**	**2.3**
			Zhang Y, 2011 [31]	TT (Ref.c)	966 (75.1)	1.00	1	
				TG	293 (22.8)	**1.54**	**1.03**	**2.31**
				GG	28 (2.1)	0.82	0.20	3.35
				TG + GG	321 (24.9)	**1.48**	**1.00**	**2.19**
rs3827665	TSC1	9	Lee JJ, 2011 [35]	dominant (common genotype model)	NR	**3.57**	**1.41**	**9.09**
rs3892097	CYP2D6	22	Azad AK, 2012^a^ [33]	G > A	GG 281 (56); GA 198 (39); AA 25 (5)	1.09	0.78	1.51
FASLG −124 A > G (rs5030772)	FASLG	1	Lei D, 2010 [25]	AA	981 (76.3)	1		
				AG + GG	305 (23.7)	1.15	0.75	1.77
			Sun Y, 2016 [29]	AA ref. (non-OPC-HNC)	63	1		
				AG + GG (non-OPC-HNC)	19	1.6	0.9	3.0
				AA ref. (OPC-HNC)	31	1		
				AG + GG (OPC-HNC)	11	1.1	0.5	2.4
rs603965	CCND1	11	Azad AK, 2012^a^ [33]	A870G G > A	GG 138 (26); GA 258 (49); AA 132 (25)	1.06	0.82	1.38
rs6596428	CDC25C	5	Lee JJ, 2011 [35]	dominant (common genotype model)	NR	**2.08**	**1.28**	**3.45**
rs678653	CCND1	11	Azad AK, 2012^a^ [33]	G1722C C > G	GG 229 (44); GC 230 (44); CC 66 (12)	0.73	0.54	0.99
rs7040593	TSC1	9	Lee JJ, 2011 [25]	dominant (common genotype model)	NR	**3.03**	**1.37**	**6.67**
rs735482	ERCC1	19	Azad AK, 2012^a^ [33]	Lys259Thr A > C	AA 409 (78); CA 111 (21); CC 6 (1)	0.94	0.62	1.44
rs739442	TSC1	9	Lee JJ, 2011 [35]	additive	NR	**1.56**	**1.12**	**2.17**
FASLG -844C > T (rs763110)	FASL	1	Lei D, 2010 [25]	CC	511 (39.7)	1		
				CT + TT	775 (60.3)	**1.71**	**1.15**	**2.54**
			Sun Y, 2016 [29]	CC ref. (non-OPC-HNC)	26	1		
				CT + TT (non-OPC-HNC)	56	**1.7**	**1.0**	**3.0**
				CC ref. (OPC-HNC)	11	1		
				CT + TT (OPC-HNC)	31	**2.7**	**1.2**	**6.0**
rs937283	MDM2	12	Jin L, 2013 [32]	AA	343 (26.7)	1		
				AG/GG	940 (73.7)	1.2	0.8	1.8
Thr 241 Met (rs861539)	XRCC3	14	Azad AK, 2012^a^ [33]	Thr241Met C > T	GG 189 (36); AG 251 (47); AA 90 (17)	1.00	0.77	1.31
			Gal TJ, 2005 [28]	Any Met	149 (58.4)	1.62	0.98	2.67
				Thr/Met	111 (43.5)	1.38	0.81	2.37
				Met/Met	38 (14.9)	**2.65**	**1.29**	**5.45**
rs9551427	FLT3	13	Lee JJ, 2011 [35]	dominant (common genotype model)	NR	**1.92**	**1.19**	**3.13**

*Abbreviations: SNPs* Single Nucleotide Polymorphisms, *NR* Not reported, *HR* Hazard Ratio, *UCI* upper confidence interval, *LCI* lower confidence interval,* ref.* reference, *HNC* head and neck cancer

^a^additive genotype model; in bold are reported statistically significant data

**Table 3:** Associations of SNPs with second primary cancer risk in five case-control studies included in systematic review

SNP	Genes investigated	Chromosome	Author, year [Ref]	Genotype	SPC/recurrence Yes, n (%)	No, n (%)	HR	LCI	UCI	OR	LCI	UCI
rs1042891 C > T	CASP6	4	Guan X, 2013 [26]	CC	480 (44.8)	462 (43.5)	N/A	N/A	N/A	1		
				CT	453 (42.5)	473 (43.7)	N/A	N/A	N/A	0.92	0.77	1.12
				TT	132 (12.4)	137 (12.8)	N/A	N/A	N/A	0.91	0.69	1.21
				CT/TT	592 (54.9)	610 (56.5)	N/A	N/A	N/A	0.93	0.77	1.11
rs1049216 A > G	CASP3	4	Guan X, 2013 [26]	AA	581 (54.5)	542 (50.5)	N/A	N/A	N/A	1		
				AG	401 (37.6)	441 (41.0)	N/A	N/A	N/A	0.83	0.68	1.00
				GG	84 (7.9)	91 (8.5)	N/A	N/A	N/A	0.86	0.62	1.20
				AG/GG	485 (45.5)	532 (49.5)	N/A	N/A	N/A	**0.83**	**0.70**	**0.99**
rs1049253 T > C	CASP3	4	Guan X, 2013 [26]	TT	669 (62.8)	734 (68.3)	N/A	N/A	N/A	1		
				TC	346 (32.4)	306 (28.5)	N/A	N/A	N/A	**1.24**	**1.02**	**1.15**
				CC	51 (4.8)	34 (3.2)	N/A	N/A	N/A	**1.80**	**1.13**	**2.87**
				TC/CC	397 (37.2)	340 (31.7)	N/A	N/A	N/A	**1.29**	**1.07**	**1.56**
rs10787498 T > G	CASP7	10	Guan X, 2013 [26]	TT	466 (43.9)	470 (43.9)	N/A	N/A	N/A	1		
				GT	482 (45.4)	486 (45.4)	N/A	N/A	N/A	1.03	0.85	1.24
				GG	114 (10.7)	115(10.7)	N/A	N/A	N/A	0.93	0.69	1.26
				GT/GG	596 (56.1)	601 (56.1)	N/A	N/A	N/A	1.01	0.84	1.20
rs11047917	KRAS	12	Wu X, 2009 [36]	C > T	123/24/0	271/22/0	**2.12**	**1.36**	**3.31**	N/A	N/A	N/A
rs11061209	RAN	12	Zhang X, 2010 [38]	AA+AG	123 (32.2)	259 (67.8)	1			N/A	N/A	N/A
				GG	24 (41.4)	34 (58.6)	**1.6**	**1.02**	**2.49**	N/A	N/A	N/A
rs1127687 G > A	CASP7	10	Guan X, 2013 [26]	GG	613 (57.5)	635 (59.1)	N/A	N/A	N/A	1		
				AG	407 (38.2)	381 (35.5)	N/A	N/A	N/A	1.09	0.9	1.31
				AA	46 (4.3)	58 (5.4)	N/A	N/A	N/A	0.8	0.52	1.21
				AG/AA	453 (42.5)	439 (40.9)	N/A	N/A	N/A	1.05	0.88	1.26
rs11586945	RGS5	1	Wang J, 2010 [24]	GG	96 (65.3)	206 (70.3)	1			N/A	N/A	N/A
				GC	41 (27.9)	76 (25.9)	1.11	0.77	1.62	N/A	N/A	N/A
				CC	10 (6.8)	11 (3.7)	**2.06**	**1.06**	**3.99**	N/A	N/A	N/A
rs11594179	SUFU	10	Zhang X, 2010 [38]	GG	103 (37.4)	172 (62.5)	1			N/A	N/A	N/A
				AG + AA	44 (26.7)	121 (73.3)	**0.67**	**0.47**	**0.96**	N/A	N/A	N/A
				AA	838 (78.1)	827 (77.1)	N/A	N/A	N/A	1		
rs12247479 A > G	CASP7	10	Guan X, 2013 [26]	AG	219 (20.6)	228 (21.2)	N/A	N/A	N/A	0.95	0.76	1.18
				GG	14 (1.3)	18 (1.7)	N/A	N/A	N/A	0.68	0.32	1.41
				AG/GG	233 (21.9)	246 (22.9)	N/A	N/A	N/A	0.93	0.75	1.15
rs12359892	MKI67	10	Wu X, 2009 [36]	T > C	101/14/27	221/38/18	**2.65**	**4.11**	**1.72**	N/A	N/A	N/A
rs12888332	MNAT1	14	Wu X, 2009 [36]	T > G	125/19/3	280/10/3	**2.57**	**1.62**	**4.09**	N/A	N/A	N/A
rs15864	GSTM3	1	Zhang X, 2010 [38]	GG + GC	127 (35.2)	234 (64.8)	1			N/A	N/A	N/A
				CC	8 (20.0)	32 (80.0)	0.49	0.24	1.00	N/A	N/A	N/A
rs17387169	PROM1	4	Wu X, 2009 [36]	G > A	129/19/2	202/85/6	**0.42**	**0.26**	**0.67**	N/A	N/A	N/A
rs17410035	RNASEN	5	Zhang X, 2010 [38]	CC + AC	141 (35.7)	254 (64.3)	1			N/A	N/A	N/A
				AA	6 (13.6)	38 (86.4)	**0.36**	**0.16**	**0.83**	N/A	N/A	N/A
rs1884056	BCL2L2	14	Zhang X, 2010 [38]	GG	59 (28.1)	151 (71.9)	1			N/A	N/A	N/A
				AG	63 (36.2)	111 (63.8)	1.41	0.98	2.03	N/A	N/A	N/A
				AA	25 (44.6)	31 (55.4)	**1.85**	**1.14**	**3.01**	N/A	N/A	N/A
rs2179653	RGS2	1	Wang J, 2010 [24]	GG	103 (70.1)	208 (70.9)	1			N/A	N/A	N/A
				GA	34 (23.1)	81 (27.6)	0.97	0.65	1.44	N/A	N/A	N/A
				AA	10 (6.8)	4 (1.4)	**2.93**	**1.50**	**5.73**	N/A	N/A	N/A
rs2227301	XPO5	6	Zhang X, 2010 [38]	GG+ AG	136 (32.6)	281 (67.4)	1			N/A	N/A	N/A
				AA	11 (47.8)	12 (52.2)	**1.91**	**1.02**	**3.58**	N/A	N/A	N/A
rs2237724	CFTR	7	Wu X, 2009 [36]	G > A	92/45/10*	198/89/6*	**3.12**	**1.62**	**5.98**	N/A	N/A	N/A
rs2300181	CAT	11	Wu X, 2009 [36]	G > A	76/58/13*	163/124/6*	**2.67**	**1.49**	**4.78**	N/A	N/A	N/A
rs2306536	CHFR	12	Wu X, 2009 [36]	G > A	87/49/11*	88/97/8*	**3.14**	**1.65**	**5.99**	N/A	N/A	N/A
rs359974	NHEJ1	2	Wu X, 2009 [36]	T > C	93/44/10*	213/76/4*	**4.26**	**2.18**	**8.32**	N/A	N/A	N/A
rs3732360	NR1I2	3	Zhang X, 2010 [38]	AA	63 (28.8)	156 (71.2)	1			N/A	N/A	N/A
				AG	67 (36.6)	116 (63.4)	1.29	0.91	1.83	N/A	N/A	N/A
				GG	17 (44.7)	21 (55.3)	**1.96**	**1.12**	**3.44**	N/A	N/A	N/A
rs3747238	SMC1B	22	Zhang X, 2010 [38]	AA+AG	108 (31.1)	239 (68.8)	1			N/A	N/A	N/A
				GG	39 (41.9)	54 (58.1)	**1.74**	**1.19**	**2.54**	N/A	N/A	N/A
rs3747813	RGS3	9	Wang J, 2010 [24]	GG	136 (92.5)	258 (88.1)	1			N/A	N/A	N/A
				GA	11 (7.4)	34 (11.6)	0.54	0.29	1.02	N/A	N/A	N/A
				AA	0	1 (0.3)	_			N/A	N/A	N/A
rs3792830	RNASEN	5	Zhang X, 2010 [38]	TT	126 (31.9)	269 (68.1)	1			N/A	N/A	N/A
				TC + CC	21 (46.7)	24 (53.3)	**1.75**	**1.08**	**2.82**	N/A	N/A	N/A
rs3795617	RGS13	1	Wang J, 2010 [24]	GG	42 (28.6)	87 (29.7)	1			N/A	N/A	N/A
				GA	80 (54.4)	119 (40.6)	1.18	0.81	1.72	N/A	N/A	N/A
				AA	25 (17.0)	87 (29.7)	**0.58**	**0.35**	**0.96**	N/A	N/A	N/A
rs3805500	RNASEN	5	Zhang X, 2010 [38]	CC	49 (25.8)	141 (74.2)	1			N/A	N/A	N/A
				CT	69 (37.7)	114 (62.3)	**1.52**	**1.05**	**2.20**	N/A	N/A	N/A
				TT	22 (44.0)	28 (56.0)	**1.91**	**1.13**	**3.23**	N/A	N/A	N/A
rs3826537	MRC2	17	Wu X, 2009 [36]	A > G	67/63/17*	88/150/55*	**0.57**	**0.41**	**0.79**	N/A	N/A	N/A
rs3917328	IL1R1	2	Zhang X, 2010 [38]	GG	131 (32.6)	271 (67.4)	1			N/A	N/A	N/A
				AG + AA	16 (42.1)	22 (57.9)	**1.8**	**1.05**	**3.07**	N/A	N/A	N/A
rs4353229 T > C	CASP7	10	Guan X, 2013 [26]	TT	611 (57.4)	614 (57.2)	N/A	N/A	N/A	1		
				CT	387 (36.4)	403 (37.5)	N/A	N/A	N/A	1.03	0.86	1.25
				CC	66 (6.2)	57 (5.3)	N/A	N/A	N/A	1.22	0.82	1.78
				CT/CC	453 (42.6)	460 (42.8)	N/A	N/A	N/A	1.06	0.88	1.26
rs506008	GSTM4	1	Wu X, 2009 [36]	G > A	94/46/3*	232/54/1*	**2.09**	**1.46**	**3.00**	N/A	N/A	N/A
rs604337	GSTM4	1	Wu X, 2009 [36]	C > T	94/50/3*	223/64/6*	**1.84**	**1.29**	**2.61**	N/A	N/A	N/A
rs639174	RNASEN	5	Zhang X, 2010 [38]	CC + CT	132 (32.4)	276 (67.6)	1			N/A	N/A	N/A
				TT	15 (48.4)	16 (51.6)	**1.88**	**1.09**	**3.25**	N/A	N/A	N/A
rs6670735	RGS8	1	Wang J, 2010 [24]	AA	68 (46.3)	111 (38.0)	1			N/A	N/A	N/A
				AG	58 (39.5)	147 (50.3)	**0.64**	**0.45**	**0.92**	N/A	N/A	N/A
				GG	21 (14.3)	34 (11.6)	0.86	0.52	1.42	N/A	N/A	N/A
rs6684195	RNF2	1	Wu X, 2009 [36]	A > G	50/64/33*	111/150/32*	**2.12**	**1.43**	**3.14**	N/A	N/A	N/A
rs6689169	RGS7	1	Wang J, 2010 [24]	AA	120 (81.6)	211 (72.0)	1			N/A	N/A	N/A
				AG	24 (16.3)	79 (26.9)	**0.6**	**0.38**	**0.94**	N/A	N/A	N/A
				GG	3 (2.0)	3 (1.0)	1.65	0.52	5.24	N/A	N/A	N/A
rs669702	RNASEN	5	Zhang X, 2010 [38]	GG	112 (31.5)	243 (68.4)	1			N/A	N/A	N/A
				GA + AA	35 (41.2)	50 (58.8)	**1.53**	**1.04**	**2.26**	N/A	N/A	N/A
rs6884823	RNASEN	5	Zhang X, 2010 [38]	GG	120 (31.5)	261 (68.5)	1			N/A	N/A	N/A
				GA + AA	27 (45.7)	32 (54.3)	**1.71**	**1.11**	**2.65**	N/A	N/A	N/A
rs699937	XPO5	6	Zhang X, 2010 [38]	CC + CT	127 (32.2)	267 (67.7)	1			N/A	N/A	N/A
				TT	20 (43.5)	26 (56.5)	**1.71**	**1.06**	**2.77**	N/A	N/A	N/A
rs7118388	CAT	11	Wu X, 2009 [36]	G > A	51/66/30*	70/139/84*	**0.67**	**0.53**	**0.84**	N/A	N/A	N/A
rs7168671	IGF1R	15	Wu X, 2009 [36]	C > T	94/44/9*	194/93/6*	**3.98**	**1.87**	**8.44**	N/A	N/A	N/A
rs7210080	SSTR2	17	Zhang X, 2010 [38]	AA+AG	133 (32.2)	280 (67.8)	1			N/A	N/A	N/A
				GG	14 (51.8)	13 (48.2)	**1.88**	**1.07**	**3.31**	N/A	N/A	N/A
rs739999	RGS11	16	Wang J, 2010 [24]	AA	113 (77.4)	242 (82.6)	1			N/A	N/A	N/A
				AG	28 (19.2)	47 (16.0)	1.16	0.76	1.77	N/A	N/A	N/A
				GG	5 (3.4)	4 (1.4)	**2.99**	**1.10**	**8.13**	N/A	N/A	N/A
rs7561607	GLI2	2	Wu X, 2009 [36]	C > T	26//82/39*	103/128/62*	**2.3**	**1.49**	**3.55**	N/A	N/A	N/A
rs7591	AXIN1	16	Wu X, 2009 [36]	A > T	33/93/21*	110/124/59*	**2.03**	**1.37**	**3.01**	N/A	N/A	N/A
rs7719666	RNASEN	5	Zhang X, 2010 [38]	TT	46 (39.3)	71 (60.7)	1			N/A	N/A	N/A
				CT	73 (34.6)	138 (65.4)	0.87	0.60	1.27	N/A	N/A	N/A
				CC	28 (25.0)	84 (75.0)	**0.59**	**0.37**	**0.95**	N/A	N/A	N/A
rs7735863	RNASEN	5	Zhang X, 2010 [38]	AA	111 (31.1)	246 (68.9)	1			N/A	N/A	N/A
				AG + GG	36 (43.4)	47 (56.6)	**1.72**	**1.16**	**2.57**	N/A	N/A	N/A
rs7781436	CDK6	7	Wu X, 2009 [36]	T > C	97/41/9*	186/104/3*	**4.51**	**2.22**	**9.17**	N/A	N/A	N/A
rs876435	TNFRSF10B	8	Wu X, 2009 [36]	A > G	51/63/33*	114/148/31*	**2.26**	**1.52**	**3.36**	N/A	N/A	N/A
rs9562605	BRCA2	13	Wu X, 2009 [36]	C > T	107/36/4*	168/124/6*	**0.54**	**0.37**	**0.78**	N/A	N/A	N/A
rs9622978	PDGFB	22	Wu X, 2009 [36]	G > T	49/63/35*	98/160/35*	**1.92**	**1.3**	**2.85**	N/A	N/A	N/A
GPX1 ALA	GPX1	3	Jefferies S, 2005 [42]	ALA5/ALA5	7 (11.2)	NR	N/A	N/A	N/A	0.46	0.20	1.07
				ALA5/ALA6	15 (23.8)	NR	N/A	N/A	N/A	0.78	0.41	1.48
				ALA5/ALA7	19 (30.1)	NR	N/A	N/A	N/A	1.43	0.78	2.63
				ALA6/ALA6	5 (7.9)	NR	N/A	N/A	N/A	0.93	0.34	2.56
				ALA6/ALA7	15 (23.8)	NR	N/A	N/A	N/A	**2.07**	**1.05**	**4.10**
				ALA7/ALA7	2 (3.2)	NR	N/A	N/A	N/A	0.57	0.13	2.57

*Abbreviations: SNP* Single Nucleotide Polymorphisms, *SPC* Second primary cancer, *NA* Not applicable, *NR* Nor reported, *HR* Hazard Ratio, *OR* Odds Ratio, *UCI* upper confidence interval, *LCI* low confidence interval, * common homozygous/ heterozygous/rare homozygous genotype; in bold are reported statistically significant data

The *p53* gene rs1042522 polymorphism was explored by four studies, of which three reported significant association for increased SPC risk. Patients with *p53* WP + PP genotype [27] and patients with *p53* 72 Arg/Pro genotype [23] had an increased SPC risk. Patients with the combined *p53* variant (Arg/Pro + Pro/Pro) genotypes, and patients with the combined polymorphisms *p53* codon 72 and *p73* G4C14-to-A4T14 [27], had a significantly increased SPC risk.

The *p21* gene C98A and C70T polymorphisms genotypes were distributed significantly different between patients who developed SPC and those who did not. Patients with p21 98 CA/AA and p21 70 CT/TT variant genotypes had a significantly increased SPC risk [30, 43]. The combined effect of both p21 polymorphisms together on SPC risk, showed that patients with either variant allele (*p21* 98 A or *p21* 70 T) had a 2-fold increased SPC risk compared with patients with the combined *p21* 98 CC and *p21* 70 CC wild-type genotypes [30, 43].

The *FAS* gene polymorphism showed that the SPC risk differed according to the index HNC site. Patients with index OPC and *FASL* 844 CT/TT genotype had significantly increased SPC risk (aHR, 2.7; 95% CI: 1.2–6.0, *p* = 0.032) while index non-OPC patients with FAS670 AG/GG and FasL844 CT/TT genotypes had significantly increased risk of SPC (aHR, 2.4 and 1.7; 95% CI, 1.1–5.1 and 1.0–3.0; and *p* = 0.043 and 0.049, respectively). Patients carrying more *FAS/FASL* variants had significantly increased risk of SPC among index non-OPC patients [29]. Overall, a non-site-specific increased SPC risk in patients with *FAS* -670 AA and the *FASLG* -844 CC genotypes was also reported by Lei D et al. [25], which further showed an increased SPC risk, in a dose-response manner, with the combined risk genotypes, of four polymorphisms on the SPC risk.

The *XPD* gene Lys751Gln polymorphism carriers had a non-significant reduced SPC risk [28, 39]. The study by Zafereo et al. [39] did not find a significant SPC risk also in stratified analysis by SPC type. However, when they combined *XPD* gene Lys751Gln polymorphism with other six SNPs of the core genes in the nucleotide excision repair (NER), in a dominant model there was a trend for increased SPC risk with increasing number of risk genotypes [39].

### Quality assessment

Results of the quality assessment of the included studies are reported in Additional file 2. Among fifteen studies without group control, only the study by Wang et al. [24], had moderate quality, while the others were of good quality. The study by Wang et al. [24] had moderate quality due to the scarcity in disclosure of potential sources of bias and for not testing of assumption and inferences for genetic analyses. Six studies with group control were of moderate quality, mostly because of the poor non-technical classification of the exposure, and for not testing of assumption and inferences for genetic analyses.

### Meta-analysis

Seven cohort studies, that reported the same genotype of the same SNP in at least two studies, were included in the meta-analysis. The included SNPs and their genotypes were *p21* C70T, CT + TT genotype; *FASLG* -844C > T, CT + TT genotype; *P21* C98A, CA + AA genotype; *FAS* gene -670A > G; *GST*-M1, Null genotype; *GST*-T1, Null genotype; and *XPD* Lys 751 Gln, Gln/Gln genotype.

All the studies included in the meta-analysis adjusted the estimates for age, sex, smoking and alcohol [25, 29, 30, 34, 39, 40, 43]. Some of the studies adjusted also for other variables: ethnicity [25, 29, 30, 39, 40, 43], treatment [29, 34] and primary HNC site [28, 29, 34].

**Fig. 2:**
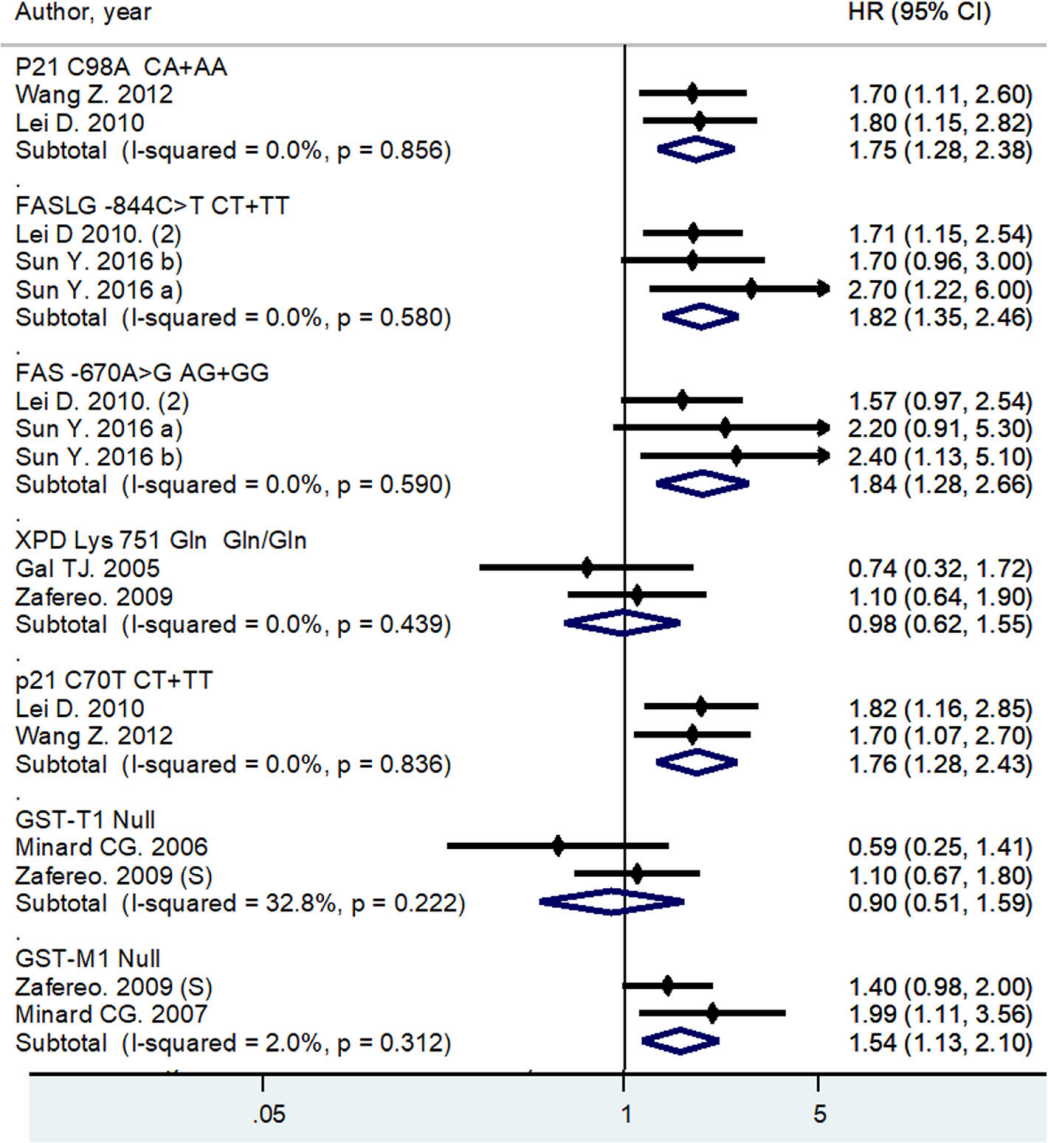
Forest plot on associations of seven Single Nucleotide Polymorphisms with Second primary cancer risk in HNC patients

The associations between SNPs and SPC risk, stratified by SNP, are shown in the Forest plot in Fig. 2. Pooled analysis revealed five SNPs with a statistically significant increased SPC risk: p21C70T, CT + TT genotype (HR = 1.76; 95% CI: 1.28–2.43; *I*^2^ = 0.0%); *FASLG* -844C > T, CT + TT genotype (HR = 1.82; 95% CI: 1.35–2.46; *I*^2^ = 0.0%); *P21* C98A, CA + AA genotype variant (HR = 1.75; 95% CI: 1.28–2.38; *I*^2^ = 0.0%); *FAS* -670A > G (HR = 1.84; 95%CI: 1.28–2.66; *I*^2^ = 0.0%) and *GST*-M1, Null genotype variant (HR = 1.54; 95% CI: 1.13–2.10; *I*^2^ = 2.0%). A non-significant decreased SPC risk was associated with *GST*-T1, Null genotype variant (HR = 0.90; 95% CI: 0.51–1.59; *I*^2^ = 32.8%); and *XPD* Lys751Gln, Gln/Gln genotype variant (HR = 0.98; 95% CI: 0.62–1.55; *I*^2^ = 0.0%). In each of the pooled analysis there was no heterogeneity between studies. The Egger test demonstrated no statistical evidence of publication bias for funnel plot FASLG -844C > T (*p* = 0.363) and FAS -670A > G (*p* = 0.24).

Stratified analysis according to the SPC site was possible for three SNPs: XPD Lys 751 Gln/Gln; GST-T1 Null; and GST-M1 Null. There was a non-significant decreased risk for HNC squamous cell carcinoma SPC in patients with XPD Lys751Gln, Gln/Gln genotype (HR = 0.46; 95% CI: 0.17–1.25; *I*^2^ = 25.1%). For tobacco-related SPC, the risk was decreased in GST-T1 Null carriers (HR = 0.83; 95% CI:0.48–1.43; *I*^2^ = 0.0%). and increased in GST-M1 Null carriers (HR = 1.53; 95% CI:0.96–2.44; *I*^2^ = 18.4%), although the associations did not reach the level of significance (Additional file 3).

## Discussion

This systematic review on genetic associations of SPC development after primary HNC included twenty-one studies and reported fifty-one genes with a significantly increased SPC risk. These genes were involved in different carcinogenesis pathways, including detoxification, DNA repair, apoptosis or cell cycle regulation, developmental pathway and cell adhesion. A total of 122 SNPs were investigated, of which eighty-one SNPs were significantly associated with an increased risk of SPC among HNC patients. Five SNPs (*p21*C70T, *FASLG* -844C > T, *P21* C98A *GST-*M1 and *GST*-T1) were significantly associated with an increased SPC risk in our meta-analysis. Genes of these five SNPs are involved in different pathways of carcinogenesis, such as apoptosis process or cell cycle regulation, DNA repair mechanism and carcinogen detoxification processes [45, 46].

*The p21 gene C98A polymorphism*, that resulted with a significantly increased SPC risk in our meta-analysis, causes a non-synonymous serine-to-arginine substitution at codon 31 and has been reported to contribute to genetic susceptibility to cancer, including HNC [47], endometrial [48] and breast cancer [49]. The influence of *P21* gene polymorphisms in cancer risk, including HNC, has been reported previously [47, 50, 51].

*FAS -670A > G* and *FASLG -844 T/C polymorphisms* have been previously associated with an increased risk of HNC [52], gynecological cancer [53] and esophageal squamous cell cancer [54], whereas *FAS* -670A > G has been also associated with an increased risk of recurrence and death in epithelial ovarian cancer patients [53]. *FAS* and *FASLG* genes have a crucial role in apoptosis processes and their polymorphisms have been reported to affect the risk of cancer, including SPCs [55]. These polymorphisms modified the risk of SPCs differently for index OPC from non-OPC patients [29], suggesting that primary cancer site may contribute to the association of apoptosis and SPC risk. The significant associations of FAS/FASL variant genotypes with the increased SPC risk in those HNC patients ever smokers or ever drinkers might suggest that genetic factors, within the context of previous or continued exposure to smoking and alcohol consumption may affect the risk of SPC development [29].

*GST-M1 null genotypes and GST-M1 null genotypes* have been previously associated with an increased risk of HNC [56] and lung cancer [57], respectively. GSTs genes, having a crucial role in carcinogen detoxification, have been associated with an increased risk of HNC, skin, breast, lung and bladder [58]. The *XPD* *Gln751Lys* polymorphism had no significant association with a reduced SPC risk from our meta-analysis, despite the fact that *XPD* gene, acting as a key DNA repair protein in the NER pathway, is involved in cancer pathogenesis.

Despite the abundant evidence about the SNPs associated with the risk of primary HNC and the biological pathways, their genetic associations with the SPC risk and the carcinogenesis pathways, in particular in HNC patients, are still not sufficiently explored, and none GWAS has addressed this susceptibility. To our knowledge, there were not publicly available data from GWAS on the genetic associations of the seven SNP included in our meta-analysis with the development of any SPC.

There are some limitations in our study that should be pointed out. We included only studies published in English, therefore some studies in other languages or currently unpublished data might have been missed, indicating the possibility of publication bias. The majority of studies included in the systematic review investigated different SNPs in the same cohort of patients, thus the SNPs included in the meta-analysis were identified by only two primary studies. The stratified analysis according to smoking, alcohol status, treatment, or primary HNC site, were conducted in only few studies, that were not the ones included in our meta-analysis. The estimates of association may be biased due to the lack of this information.

Although data were pooled in the meta-analysis according to the genotype variant, it was not possible to identify the best genetic model for each SNP, because not all the studies reported genotype data that could enable us to calculate the estimates according to a genetic model. The results may vary depending on the genetic model used in statistical significance, however still remains the uncertainty on the best genetic model for the association of interest.

Despite these limitations, we addressed the potential publication bias by exploring GWAS publicly available websites. Except one study of moderate quality, the other studies included in the systematic review, and also in the meta-analysis were of good quality. To our knowledge, this is the first systematic review and meta-analysis to summarize the studies on the genetic associations of SPC development after primary HNC. The large number of genes included in this systematic review, could serve as an import guide to the researchers to choose the genes that can be studied further in the future. However, the reported associations, in particular for the polymorphisms not included in the meta-analysis, need to be confirmed in future studies.

Studies included in the systematic review reported small number of HNC patients developing SPC due to the insufficient follow-up times. Therefore, GWASs, larger and well-designed studies with longer follow-up time, and further studies pathway-oriented on biological functions of the polymorphisms, are needed in order to improve our knowledge of the genetic associations that influence the SPC occurrence after a primary HNC. Furthermore, the combined effect of a panel of polymorphisms that act in the same carcinogen metabolizing pathway support the notion that SPC development after a primary HNC is a polygenic process. The effects of associations of these polymorphisms with the SPC risk might be amplified, suggesting that their further exploration may provide higher predictive estimates of association [36, 40]. In HNC patients, genetic testing for these SNPs, with evidences on clinical validity and utility, might be helpful for the identification of high-risk patients for developing a SPC, leading to personalized approaches and an early diagnosis of SPC. Moreover, considering that both, HNC and SPC result from complex interactions of genetic variants and environmental factors, further studies should focus on these interactions in SPC development. Future studies should also investigate the influence of risk factors, such as tobacco smoking, alcohol consumption or HPV status, and different treatment modalities in overall and site-specific SPC risk.

## Conclusions

The polymorphisms identified and summarized in this study may serve as a potential therapeutic targets or markers for genetic susceptibility to SPC after an index HNC, that may further enhance the identification of high-risk groups of HNC patients, aiming to provide a personalized treatment for an improved prognosis and a better quality of life.

## Supplementary Information

**Additional file 1.**  Preferred Reporting Items for Systematic Reviews and Meta-Analyses (PRISMA) Checklist.

**Additional file 2.** Quality assessment of the twenty-one studies included in the systematic review using Q-Genie Tool.

**Additional file 3.** Stratified analyses according to Second Primary Cancer site, for each SNP genotype.

## Data Availability

The datasets used and/or analyzed during the current study are available from the corresponding author on reasonable request.

## Abbreviations

HNC: Head and Neck Cancer
SPC: Second Primary Cancer
CI: Confidence Interval
HR: Hazard Ratios
OR: Odds Ratio
GWAS: Genome wide association studies
SNPs: Single nucleotide polymorphisms
OPC: Oropharyngeal cancer
