# Supplementary material for: Single nucleotide polymorphisms and the risk of developing a second primary cancer among head and neck cancer patients: a systematic literature review and meta-analysis
Source: BMC Cancer. 2021 Jun 2;21:660. doi: 10.1186/s12885-021-08335-0 (PMC8173958; doi:10.1186/s12885-021-08335-0)
Supplement: Supplementary file 2 — Additional file 2. Quality assessment of the twenty-one studies included in the systematic review using Q-Genie Tool. [file 12885_2021_8335_MOESM2_ESM.docx]

**Supplementary File 2.** Quality assessment of the twenty-one studies included in the systematic review using Q-Genie Tool

| **Author, year [Ref]** | **Rationale** | **Selection and definition of outcome of interest** | **Selection and comparability of outcome of interest** | **Technical classification of the exposure** | **Non-technical classification of the exposure** | **Other source of bias** | **Sample size and power** | **A priori planning of the analysis** | **Statistical methods and control for confounding** | **Testing of assumption and inferences for genetic analyses** | **Appropriateness of inferences drawn from results** | **Total** |
| --- | --- | --- | --- | --- | --- | --- | --- | --- | --- | --- | --- | --- |
| Leoncini E, 2015 [30] | 6 | 6 | N/A | 5 | 4 | 5 | 3 | 6 | 5 | 2 | 6 | 48 |
| Jin L, 201 | 4 | 6 | N/A | 3 | 5 | 1 | 2 | 5 | 2 | 1 | 4 | 33 |
| Lei D, 2010 [33] | 7 | 6 | N/A | 5 | 6 | 4 | 4 | 7 | 5 | 2 | 6 | 52 |
| Wang Z, 2012 [38] | 6 | 6 | N/A | 5 | 5 | 5 | 4 | 5 | 5 | 2 | 6 | 49 |
| Azad AK, 2012 [41] | 5 | 5 | N/A | 4 | 4 | 5 | 4 | 6 | 5 | 5 | 6 | 49 |
| Lei D, 2010 [32] | 6 | 6 | N/A | 5 | 5 | 5 | 4 | 6 | 4 | 2 | 6 | 49 |
| Minard, 2006 [42] | 4 | 5 | N/A | 3 | 4 | 4 | 2 | 7 | 5 | 1 | 6 | 41 |
| Gal TJ, 2005 [36] | 5 | 3 | N/A | 2 | 5 | 6 | 2 | 6 | 5 | 1 | 7 | 42 |
| Sun Y, 2016 [37] | 6 | 6 | N/A | 5 | 5 | 6 | 4 | 6 | 3 | 2 | 6 | 49 |
| Zhang Y, 2011[39] | 6 | 6 | N/A | 5 | 5 | 5 | 4 | 6 | 5 | 1 | 6 | 49 |
| Zhang Y, 2012 [35] | 6 | 6 | N/A | 5 | 5 | 5 | 4 | 6 | 5 | 2 | 6 | 50 |
| Guan X, 2013* [34] | 6 | 6 | 4 | 5 | 5 | 4 | 4 | 5 | 5 | 2 | 6 | 52 |
| Li F. 2010  [23] | 6 | 6 | N/A | 6 | 5 | 5 | 4 | 6 | 3 | 2 | 6 | 49 |
| Wu X. 2009* [43] | 6 | 5 | 5 | 4 | 3 | 3 | 4 | 5 | 5 | 1 | 5 | 46 |
| Wang J, 2010* [24] | 6 | 6 | 4 | 3 | 2 | 3 | 3 | 5 | 5 | 2 | 5 | 44 |
| Lee JJ, 2011* [25] | 6 | 6 | 6 | 4 | 3 | 3 | 4 | 5 | 2 | 2 | 6 | 47 |
| Li F. 2009  [26] | 6 | 6 | N/A | 5 | 6 | 4 | 4 | 6 | 1 | 2 | 6 | 46 |
| Zhang X, 2010* [27] | 5 | 5 | 4 | 3 | 2 | 1 | 3 | 4 | 3 | 1 | 5 | 36 |
| Zafereo E, 2009 [28] | 6 | 6 | N/A | 5 | 5 | 5 | 4 | 6 | 3 | 1 | 6 | 47 |
| Zafereo, 2009 [29] | 6 | 6 | N/A | 5 | 5 | 5 | 4 | 6 | 3 | 1 | 6 | 47 |
| Jefferies S, 2005* [31] | 5 | 4 | 4 | 3 | 3 | 3 | 3 | 3 | 2 | 1 | 5 | 36 |

*Abbreviations:* N/A -Not applicable, *-studies with control group
