# Supplementary material for: Single nucleotide polymorphisms and the risk of developing a second primary cancer among head and neck cancer patients: a systematic literature review and meta-analysis
Source: BMC Cancer. 2021 Jun 2;21:660. doi: 10.1186/s12885-021-08335-0 (PMC8173958; doi:10.1186/s12885-021-08335-0)
Supplement: Supplementary file 3 — Additional file 3. Stratified analyses according to Second Primary Cancer site, for each SNP genotype. [file 12885_2021_8335_MOESM3_ESM.docx]

|  | **HNSCC SPC** | |  |
| --- | --- | --- | --- |
| **XPD Lys 751 Gln/Gln** |  |  |  |
| Study | HR | 95% CI | % Weight |
| Gal TJ. 2005 | 0.17 | 0.02-1.29 | 20.23 |
| Zafereo. 2009 | 0.60 | 0.30-1.19 | 79.77 |
| Subtotal (I-squared=25.1%, p=0.625) | 0.46 | 0.17-1.25 | 100.00 |
|  |  |  |  |
|  | **Tobacco-related SPC** | |  |
| **GST-T1 Null** |  |  |  |
| Study | HR | 95% CI | % Weight |
| Minard CG. 2006 | 0.66 | 0.22-1.92 | 26.18 |
| Zafereo. 2009 (S) | 0.90 | 0.47-1.70 | 73.82 |
| Subtotal (I-squared=0.0%, p=0.625) | 0.83 | 0.48-1.43 | 100.00 |
|  |  |  |  |
| **GST-M1 Null** |  |  |  |
| Study | HR | 95% CI | % Weight |
| Zafereo. 2009 (S) | 1.300 | 0.80-2.10 | 67.57 |
| Minard CG. 2007 | 2.160 | 1.01-4.62 | 32.43 |
| Subtotal (I-squared=18.4%, p=0.268) | 1.533 | 0.96-2.44 | 100.00 |

**Supplementary file 3.** Stratified analyses according to SPC site, for each genotype.
